# Chemotherapeutic Potential of 17-AAG against Cutaneous Leishmaniasis Caused by *Leishmania (Viannia) braziliensis*

**DOI:** 10.1371/journal.pntd.0003275

**Published:** 2014-10-23

**Authors:** Diego M. Santos, Antonio L. O. A. Petersen, Fabiana S. Celes, Valeria M. Borges, Patricia S. T. Veras, Camila I. de Oliveira

**Affiliations:** 1 Centro de Pesquisas Gonçalo Moniz, Fundação Oswaldo Cruz (FIOCRUZ), Salvador, Bahia, Brazil; 2 Instituto Nacional de Ciência e Tecnologia de Investigação em Imunologia (iii-INCT), Salvador, Bahia, Brazil; Louisiana State University, United States of America

## Abstract

**Background:**

Leishmaniasis remains a worldwide public health problem. The limited therapeutic options, drug toxicity and reports of resistance, reinforce the need for the development of new treatment options. Previously, we showed that 17-(allylamino)-17-demethoxygeldanamycin (17-AAG), a Heat Shock Protein 90 (HSP90)-specific inhibitor, reduces *L. (L.) amazonensis* infection *in vitro*. Herein, we expand the current knowledge on the leishmanicidal activity of 17-AAG against cutaneous leishmaniasis, employing an experimental model of infection with *L. (V.) braziliensis*.

**Methodology/Principal findings:**

Exposure of axenic *L. (V.) braziliensis* promastigotes to 17-AAG resulted in direct dose-dependent parasite killing. These results were extended to *L. (V.) braziliensis*-infected macrophages, an effect that was dissociated from the production of nitric oxide (NO), superoxide (O^−2^) or inflammatory mediators such as TNF-α, IL-6 and MCP-1. The leishmanicidal effect was then demonstrated *in vivo*, employing BALB/c mice infected with *L. braziliensis*. In this model, 17-AAG treatment resulted in smaller skin lesions and parasite counts were also significantly reduced. Lastly, 17-AAG showed a similar effect to amphotericin B regarding the ability to reduce parasite viability.

**Conclusion/Significance:**

17-AAG effectively inhibited the growth of *L. braziliensis*, both *in vitro* and *in vivo*. Given the chronicity of *L. (V.) braziliensis* infection and its association with mucocutaneous leishmaniasis, 17-AAG can be envisaged as a new chemotherapeutic alternative for cutaneous Leishmaniasis.

## Author Summary

Antimony-containing compounds are the main drugs used to treat leishmaniasis but the severe associated side effects pose the need for alternative chemotherapeutic options. Herein, we evaluated the ability of 17-AAG (a Heat Shock Protein 90 inhibitor) to kill *Leishmania (Viannia) braziliensis* parasites, a species that causes both cutaneous and mucocutaneous Leishmaniasis in Brazil. Heat Shock Protein 90 (HSP90) is associated with important biological processes; inhibition of this molecule interferes with parasite survival and, hence, it can be exploited as a chemotherapeutic target. We show that exposure to 17-AAG induced killing of *L. brazilensis* parasites in both its extracellular and intracellular forms. This effect was not dependent on the activation of the host cell. More importantly, treatment of mice infected with *L. (V.) braziliensis* also modulated lesion development and decreased parasite growth at the infection site. Collectively, our results show that targeting HSP90 is a promising alternative for development of novel chemotherapeutic options for leishmaniasis.

## Introduction

Leishmaniasis is a widespread group of parasitic diseases caused by protozoa of the genus *Leishmania*, that is transmitted by the bite of female sand flies. Currently, about 12 million people are at risk of leishmaniasis and there are an estimated 1.5–2 million new cases each year [1]. There are two main clinical manifestations: visceral leishmaniasis, affecting mainly the spleen and liver and cutaneous leishmaniasis, affecting the skin. CL caused by *Leishmania (V.) braziliensis* is particularly distinguished from other leishmaniasis by its chronicity, latency and tendency to metastasize in the human host [2]. In 1–5% of patients, mucocutaneous leishmaniasis may develop due to the ability of *L. (V.) braziliensis* to persist within lesion scars after spontaneous or chemotherapy-mediated healing and to its ability to metastasize to the nasal mucosal [3], [4]. In this case, extensive tissue destruction is observed, resulting from the potent cell-mediated immune response triggered by parasite replication [5]. More rarely, parasite invasion of the bloodstream results in disseminated skin lesions [6]. Brazil along with nine other countries account for 70–75% of the global estimated CL incidence [7].

The drugs of first choice for leishmaniasis chemotherapy are Pentavalent Antimonials (Sb^+5^) [8], which interfere with the oxidative metabolism of intracellular *Leishmania* [5], [9], [10]. These compounds are significantly toxic and have been associated with drug resistance [11], [12]. Amphotericin B and Paramomycin, two other drugs available [13]–[15], also display limitations with regards to toxicity, cost and/or duration of treatment [16]. In the current scenario, the identification of new chemotherapeutic compounds is urgently needed, especially since vaccines against leishmaniasis are not yet available.

Heat Shock Proteins (HSPs) form complexes that act as chaperones, binding other proteins, denominated client proteins. These multimolecular complexes are involved in regulating protein folding, intracellular protein transport and repair or degradation of proteins partially denatured due to stress, for example [17], [18]. Among the HSPs, HSP90 is one of the most abundant cellular chaperones and many of its client proteins are involved in cell signaling, proliferation and survival [19]. It is essential for oncogenic transformation and exploited by malignant cells to support cancer-associated kinases and transcription factors [20]. HSP90 also plays an important role in protozoans such as *Leishmania* and *Trypanosoma*, which critically rely on HSP90 for survival in alternating environments associated with their complex life cycles [21]. Therefore, HSP90-inhibitors become interesting candidates for leishmaniasis chemotherapy.

Treatment of *L. donovani* parasites with geldanamycin (GA), a HSP90-specific inhibitor, arrested promastigote growth and differentiation into amastigotes [22]. It also reduced gluthathione levels, increasing the production of reactive oxygen species (ROS) and promoting apoptosis [23]. Recently, we reported on the effects of 17-(allylamino)-17-demethoxygeldanamycin (17-AAG) on *L. (L.) amazonensis* [24]. 17-AAG is a HSP90-specific inhibitor analogous to geldanamycin (GA) [25]. Macrophages infected with *L. (L.) amazonensis* and treated with a low dose of 17-AAG displayed significantly smaller parasite loads, an effect that was not mediated by activation of the macrophage inflammatory response [24].

In the present work, we expanded our previous observations to the effects of 17-AAG on *L. (V.) braziliensis*, the etiological agent of both cutaneous and mucocutaneous leishmaniasis in Brazil. Experiments were performed *in vitro* and *in vivo*, employing an experimental model [26]. 17-AAG was efficient at reducing *L. (V.) braziliensis* promastigote growth and macrophage infection. More importantly, 17-AAG was equally efficient *in vivo*, highlighting its potential as a novel chemotherapy agent against CL caused by *L. (V.) braziliensis*.

## Methods

### Ethics statement

Female BALB/c mice, 6–8 weeks of age, were obtained from CPqGM/FIOCRUZ animal facility where they were maintained under pathogen-free conditions. All animal work was conducted according to the Guidelines for Animal Experimentation of the Colégio Brasileiro de Experimentação Animal and of the Conselho Nacional de Controle de Experimentação Animal. The local Ethics Committee on Animal Care and Utilization (CEUA) approved all procedures involving animals (CEUA-L001/12-CPqGM/FIOCRUZ).

### 17-AAG and amphotericin B

17-AAG (17-(allylamino)-17-demethoxygeldanamycin) (Invivogen) was dissolved in Dimethyl sulfoxide (DMSO) (SIGMA) to a 5 mM stock solution, stored at -20°C in aliquots. For in vitro use, the stock solution was diluted in cell culture medium to the desired concentration at the time of use. For *in vivo* treatments, a stock solution was prepared at 100 mg/ml and diluted to 20 mg/kg at the time of use. Amphotericin B (Fungizone, Life Technologies) was dissolved in DMEM medium to a 250 ug/ml stock solution. The stock solution was diluted in cell culture medium to the desired concentration at the time of use.

### Parasite culture

*L. (V.) braziliensis* (MHOM/BR/01/BA788) [26] was cultured at 26°C in Schneider's insect medium (Invitrogen) supplemented with 10% inactive Fetal Bovine Serum (FBS), 2 mM L-glutamine, 100 U/ml penicillin, and 100 mg/ml streptomycin (all from Invitrogen).

### *L. (V.) braziliensis* promastigotes viability assay

Axenic *L. (V.) braziliensis* promastigotes (1×10^6^ parasites/ml), cultivated in supplemented Schneider medium, were treated with increasing concentrations of 17-AAG (25, 75, 125, 250, 500 or 625 nM). After 48 h, parasite viability was evaluated by direct counting of live motile parasites using a Neubauer chamber. In some experiments, promastigotes were treated with the half maximal inhibitory concentration (IC_50_) (65 nM). After 48 h, promastigotes were washed three times with PBS and were further cultured for 24 and 48 h in supplemented Schneider medium, devoid of 17-AAG. The number of viable promastigotes was determined by direct counting.

### Macrophage infection with *L. (V.) braziliensis* and treatment with 17-AAG

BALB/c mice were injected i.p. with 3% thioglycolate. Five days after injection, peritoneal lavage was performed using 8 ml DMEM medium supplemented with 10% Fetal Calf Serum (FCS), 2 mM L-glutamine, 100 U/ml penicillin and 100 µg/ml streptomycin (all from Invitrogen). To obtain monolayers, cells (6×10^5^ cells/ml) were place into glass coverslips within the wells of a 24-well plate and were left to adhere for 2 h, at 37°C and 5% CO_2_. Non-adherent cells were removed by gentle and extensive washing with PBS; purity was routinely above 99%. Remaining cells (3×10^5^ cells/ml) received 3×10^6^ cells/ml of stationary-phase *L. (V.) braziliensis* promastigotes and were incubated at 37°C in supplemented DMEM medium. After 24 h of infection, glass coverslips containing infected macrophages were washed to remove non-internalized parasites and cells were treated with different concentrations of 17-AAG (25, 100, 250 and 500 nM) for 12–72 h. Control groups were incubated in supplemented DMEM medium containing DMSO only. Glass coverslips were washed and stained with H&E and the intracellular amastigotes were counted by light microscopy. The results are shown as the percentage of infected cells and the number of intracellular amastigotes was counted in 400 macrophages. Cultures were performed in quintuplicate. Alternatively, infected macrophages were washed extensively and the medium was replaced with 0.5 ml of supplemented Schneider medium, devoid of 17-AAG. Cells were cultured at 26°C for an additional 5 days and the number of viable parasites was determined by direct counting. In some experiments, infected macrophages were treated with the half maximal inhibitory concentration (IC_50_) (220 nM) of 17-AAG or with amphotericin B (0.25 µg/ml; 0.27 µM) for 24 h. Parasite viability was determined by direct counting.

### Viability of 17-AAG-treated murine macrophages

Macrophages (2×10^5^ cells/ml), obtained as above, were treated increasing concentrations of 17-AAG (39–20,000 nM) or with DMSO for 72 h. Next, cultures were washed twice cells were incubated with supplemented DMEM containing 10% AlamarBlue (Invitrogen). Cells were incubated for another 4 h and reagent absorbance was measured at the wavelengths of 570 nm and 600 nm using a spectrophotometer (SPECTRA Max 340 PC). Ethanol-fixed cells were used as positive controls.

### Detection of NO, reactive oxygen species, cytokines and chemokines

Macrophages (3×10^6^ cells/ml), obtained as above, were stimulated with IFN-γ (100 UI/ml) (Sigma) and were infected with *L. (V.) braziliensis* (3×10^7^ cells/ml) for 24 h. Macrophage cultures were then washed to remove non-internalized parasites and fresh culture medium containing IFN-γ and 220 nM of 17-AAG was added. Cultures supernatants were collected 48 h later. Griess reaction was used to measure nitric oxide (NO) production by determining concentration of its stable reaction product nitrite (NO_2_^−^) [27]. Superoxide (SO) production was determined by adding hydroxylamine (Sigma) (0.5 mM) [28], [29] to infected macrophages. Hydroxylamine converts superoxide into nitrite, which is then be quantitated by the Griess reaction, as described above. Background levels of nitrite generated by the release of NO were determined in parallel, without the addition of hydroxylamine. Production of TNF-α, IL-6, IL-10 and CCL2/MCP-1 was evaluated using an inflammatory Cytometric Bead Array (BD Biosciences) following the manufacturer's instructions. Data were acquired and analyzed using a FACSort flow cytometer and FCAP Array (V.3.0) (BD Biosciences).

Intradermal infection with *L. (V.) braziliensis and in vivo* treatment with 17-AAG

BALB/c mice were inoculated with stationary-phase *L. (V.) braziliensis* promastigotes (10^5^ parasites in 10 µl of saline) in the left ear dermis using a 27.5-gauge needle. Four weeks post-infection, mice (n = 10) were treated 3 times/wk for 3 weeks with 17-AAG (20 mg/kg of 17-AAG diluted in DMSO i.p.). The control group (n = 10) received i.p. injections of DMSO in the same concentrations used in 17-AAG treated animals (n = 10). Lesion size was monitored weekly for 10 weeks using a digital caliper (Thomas Scientific). Parasite load was determined using a quantitative limiting-dilution assay as described elsewhere [26]. Briefly, infected ears and lymph nodes draining the infection site were aseptically excised six weeks post-infection and homogenized in Schneider medium. Homogenates were serially diluted in supplemented Schneider complete and seeded into 96-well plates. The number of viable parasites was determined from the highest dilution at which the promastigotes could be grown after up to 2 weeks of incubation at 26°C.

### Statistical analysis

The half maximal inhibitory concentration (IC_50_) of 17-AAG on *L. braziliensis* promastigotes and on intracellular *L. braziliensis* amastigotes were determined from sigmoidal regression of the concentration-responses curves, respectively, using Prism (GraphPad Prism V. 6.0). The selectivity index of 17-AAG was calculated as the ratio between the CC_50_ for murine macrophages and the IC_50_ for intracellular *L. braziliensis* amastigotes.

Data are presented as the mean ± standard error of the mean. Kolmogorov-Smirnov was used for normality analysis. Parametric (One-way ANOVA followed by Dunnett's Multiple Comparison Test or post-test for linear trend or by Bonferroni) or non-parametric analysis (Mann-Whitney) tests were also performed using Prism (GraphPad software, V. 6.0) To evaluate disease burden in mice, ear thickness of mice treated with 17-AAG or DMSO was recorded weekly for each individual mouse. The course of disease for 17-AAG-treated and control mice was plotted individually, and the area under each resulting curve was calculated using a non-parametric test (Mann-Whitney). *p*-values ≤ 0.05 were considered significant.

## Results

### Exposure to 17-AAG reduces the viability of *L. (V.) braziliensis* promastigotes

**Figure 1:**
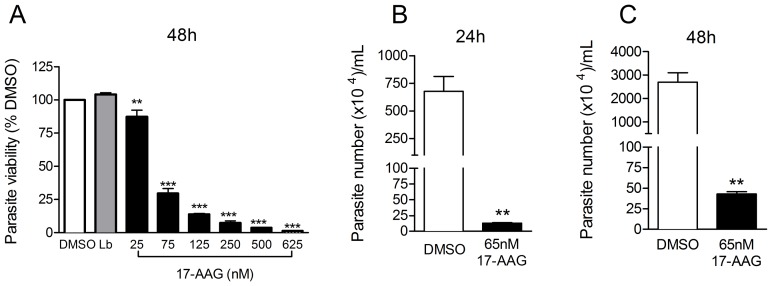
17-AAG induces killing of *Leishmania (V.) braziliensis* promastigotes in a dose-dependent and irreversible manner. *L. (V.) braziliensis* promastigotes were exposed to increasing concentrations of 17-AAG, to vehicle alone (DMSO) or were left unexposed (Lb) for 48 h. (A) The number of viable parasites was evaluated by direct counting. *L.(V) braziliensis* promastigotes were treated with 65 nM (IC_50_) of 17-AAG for 24 h (B) and (C) 48 h. After washing, promastigotes were cultured for additional 48 h and the number of viable parasites was evaluated. Data, shown as mean ±SEM, are from one of two independent repeats (**p<0.01 and ***p<0.001).

Initially, we investigated the effects of 17-AAG on axenic *L. (V.) braziliensis* promastigotes. Parasites were incubated with increasing concentrations of 17-AAG for 48 h and viability was quantified by direct counting. All 17-AAG-treated cultures showed a significantly lower number of parasites in comparison to the control, treated with vehicle (DMSO) alone (Figure 1A) (One-way ANOVA, p<0.001). After 48 h of treatment, 17-AAG (25 nM) reduced parasite viability by 13% (Figure 1A) (compared to DMSO-treated cultures) and increasing concentrations of 17-AAG (75–625 nM), maximized killing effects. At the highest concentration tested (625 nM) parasite viability was reduced by 98% when compared to DMSO-treated cultures (Figure 1A). These results also indicate that parasite viability was reduced in a dose-dependent effect (One-way ANOVA, p<0.001 followed by test for linear trend *p*<0.001). The DMSO concentration used was not toxic as parasite viability was similar in cultures left untreated (Lb) or treated with DMSO only (Figure 1A). Based on these results, IC_50_, after 48 h of 17-AAG treatment, was established at 65 nM (Figure S1). To evaluate whether the effect of 17-AAG on *L. (V.) braziliensis* promastigotes was reversible, parasites were treated with 17-AAG (65 nM) for 48 h, washed and subsequently re-incubated in 17-AAG-free medium, for an additional 24 and 48 h. Parasite numbers were significantly reduced (*p*<0.01) in cultures kept for both 24 h (Figure 1B) and 48 h (Figure 1C). These results show that the effect of 17-AAG on *L. (V.) braziliensis* promastigotes is irreversible.

### 17-AAG reduces parasite load in *L. (V.) braziliensis*-infected macrophages

**Figure 2:**
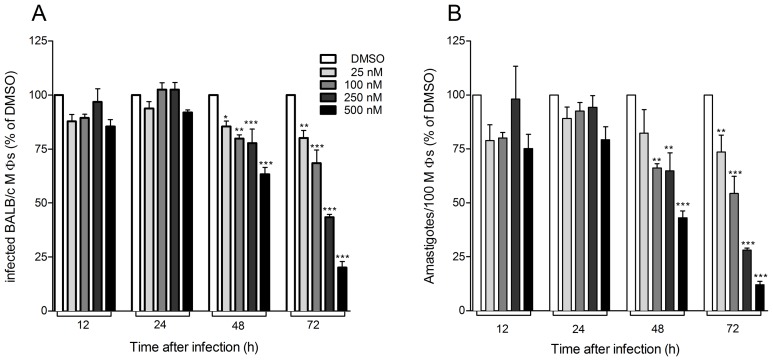
Treatment with 17-AAG controls *L. (V.) braziliensis* replication inside macrophages. *L. (V.) braziliensis-* infected macrophages were treated with increasing concentrations of 17-AAG or with vehicle alone (DMSO). After 12–72 h, glass coverslips were stained with H&E and assessed for the percentage of infected macrophages (A) and the number of amastigotes per 100 macrophages (B) by light microscopy. Data, shown as mean ±SEM, are shown as the percentage of DMSO -treated cultures, from one of three independent repeats (*p<0.05; **p<0.01 and ***p<0.001).

Next, we investigated the effect of 17-AAG on intracellular *L. (V.) braziliensis* amastigotes. BALB/c macrophages were infected with *L. (V.) braziliensis* and cells were treated with a range of 17-AAG concentrations (25–500 nM) for 12–72 h. At each time point, cells were fixed and the parasite load was assessed by light microscopy. At the initial time points (12 and 24 h), we did not detect significant alterations in treated cultures versus control cultures (DMSO-treated) (Figure 2A and B). After 48 h, 17-AAG (25 nM) reduced the infection rate to 85% (Figure 2A) and intracellular amastigotes were reduced to 82% (compared to the percentages obtained in DMSO-treated cultures) (Figure 2B). With increasing concentrations of 17-AAG (100–500 nM), these effects became more pronounced and, at 500 nM, the percentage of infected cells was reduced to 63% (Figure 2A) and of intracellular amastigotes to 43% (Figure 2B) (One-way ANOVA, p<0.001) (again compared to the percentages obtained in DMSO-treated cultures). After 72 h of treatment, these effects were maximal: 500 nM of 17-AAG decreased the infection rate to 20% (Figure 2A) whereas intracellular amastigotes were reduced to 11% (Figure 2B). The absolute percentages of infection, following treatment with different concentrations of17-AAG and the absolute numbers of amastigotes/100 cells over time are shown in Figures S2A and S2B, respectively. As with promastigotes (Figure 1A), effects observed with 17-AAG on intracellular macrophages were dose-dependent (One-way ANOVA, p<0.001 followed by test for linear trend *p*<0.001). IC_50_, after 72 h of 17-AAG treatment, was determined as 220 nM (Figure S3); additionally, 17-AAG employed at different concentrations (125, 220 and 500 nM) did not compromise macrophage viability as assayed by MTT (Figure S4). Cytotoxicity against murine macrophages was determined upon treatment of non-infected macrophage cultures with 17-AAG with a calculated CC_50_ of 3.6 nM. The selectivity index of 17-AAG was established at 16.6.

### 17-AAG reduces the viability of intracellular *L. (V.) braziliensis* amastigotes

**Figure 3:**
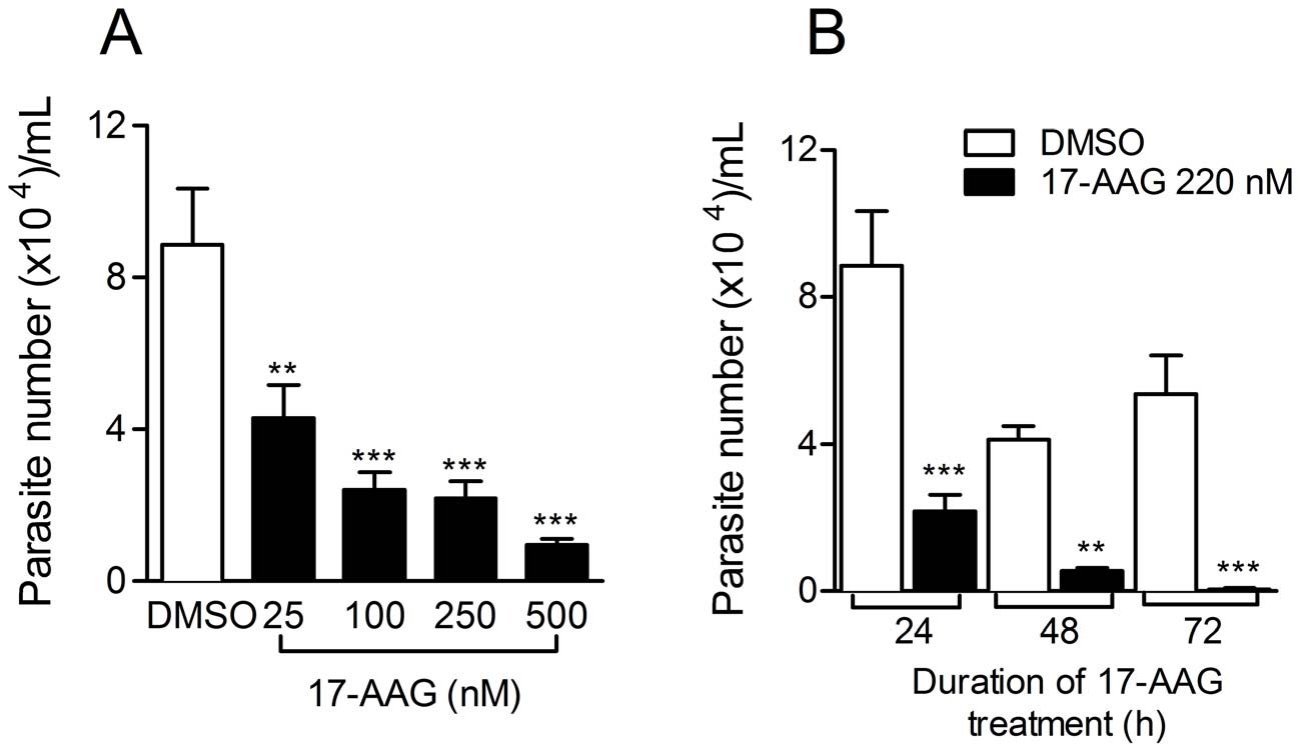
Treatment with 17-AAG reduces intracellular *L. (V.) braziliensis* viability. *L. (V.) braziliensis*- infected macrophages were treated for 24 h with increasing concentrations of 17-AAG or with vehicle alone (DMSO). The number of viable parasites was evaluated by further culture (5 days) in Schneider medium, free of 17-AAG (A). Infected macrophages were treated with 220 nM (IC_50_) of 17-AAG for 24–72 h. The number of viable parasites was evaluated by further culture for five days in Schneider medium, free of 17-AAG (B). Data, shown as mean ±SEM, are from one of two independent repeats (**p<0.01 and *** *p*<0.001).

Although 17-AAG treatment of *L. (V.) braziliensis*-infected macrophages for 24 h did not significantly modify the infection rate (Figure 2), we asked whether it would alter amastigote viability. Cells were infected and treated with a range of 17-AAG concentrations (25–500 nM). Intracellular parasite survival was determined following the replacement of DMEM for Schneider culture medium and direct counting of surviving *L. (V.) braziliensis*. Five days after medium replacement, treatment with increasing concentrations of 17-AAG for 24 h significantly reduced the number of viable *L. (V.) braziliensis* parasites (Figure 3A) (One-way ANOVA, p<0.001), indicating once more a dose-dependent effect (test for linear trend *p*<0.001). This effect was also time-dependent as exposure to 220 nM (IC_50_) of 17-AAG for longer periods (48 and 72 h) also significantly decreased the number of *L. (V.) braziliensis* promastigotes (Figure 3B). Therefore, exposure of infected macrophages to 17-AAG negatively impacted on the survival of *L. (V.) braziliensis.*

### 17-AAG down-modulates the production of inflammatory mediators by *L. (V.) braziliensis* -infected macrophages

**Figure 4:**
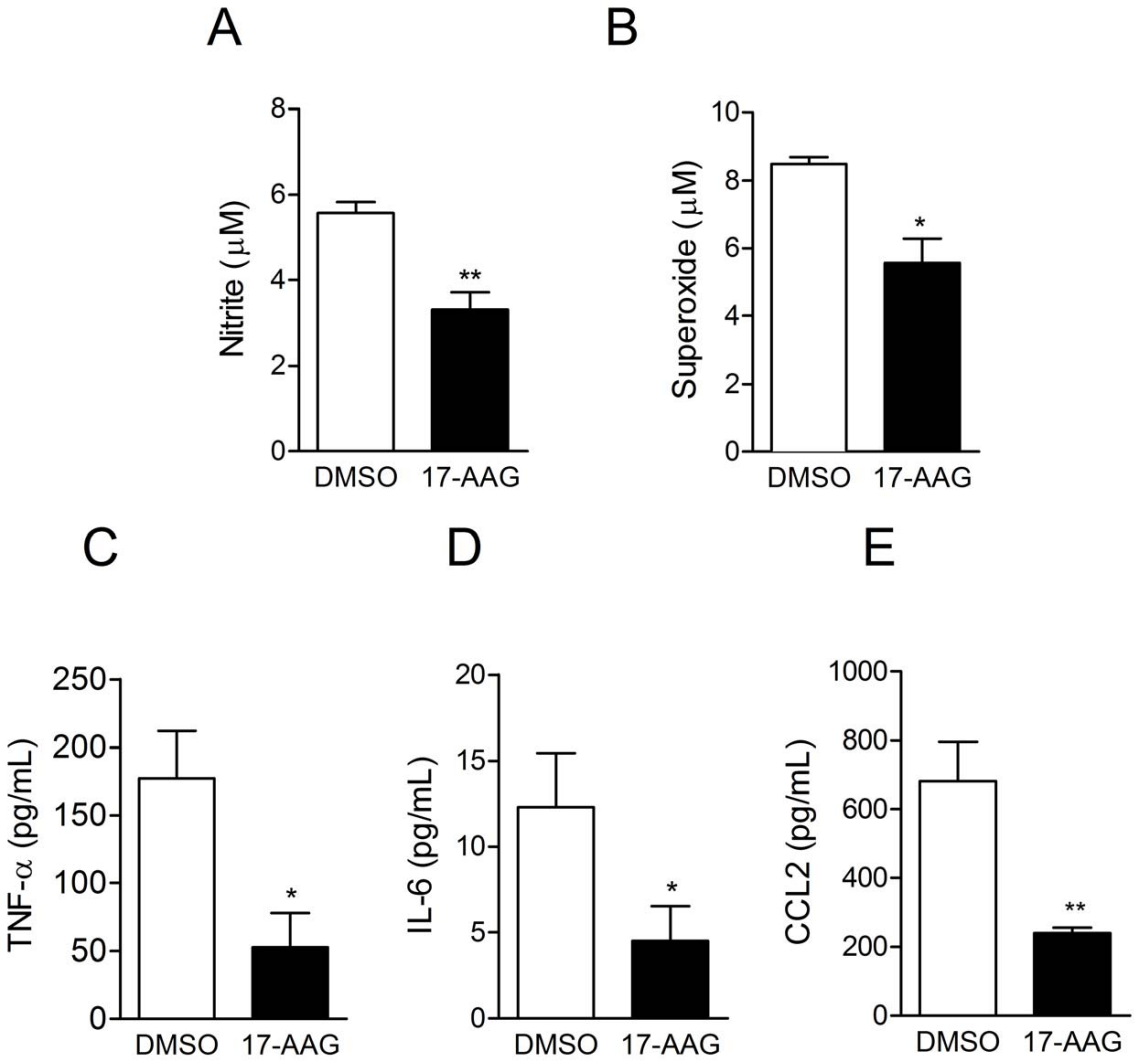
Treatment with 17-AAG down regulates ROS and cytokine production in *L. (V.) braziliensis-infected* cells. *L. (V.) braziliensis-*infected macrophages were treated with 17-AAG (220nM) + IFN-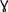. After 48h, supernatants were assayed for nitrite production (A) and for presence of (B) superoxide, following addition of hydroxylamine. The presence of secreted (C) TNF-α, (D) IL-6 and (E) CCL2 was determined in culture supernatants by Cytometric Bead Array, after 24 h of treatment. Data, shown as mean ± SEM, are from one of two independent repeats (**p<0.01; *p<0.05).

Macrophage activation and production of nitric oxide and superoxide are key steps towards elimination of intracellular *Leishmania* [30]. In macrophages infected with *L. (V.) braziliensis* and treated with 220 nM 17-AAG (IC_50_), production of nitric oxide (Figure 4A) and superoxide (Figure 4B) were lower compared to cells exposed to DMSO. 17-AAG-treatment also down-modulated the production of TNF-α (Figure 4C), IL-6 (Figure 4D) and CCL2/MCP-1 (Figure 4E) by *L. (V.) braziliensis*-infected macrophages. Therefore, the leishmanicidal effect of 17-AAG is uncoupled from the production of microbicidal molecules and from the production of pro-inflammatory cytokines.

### In vivo control of CL caused by L. (V.) braziliensis

**Figure 5:**
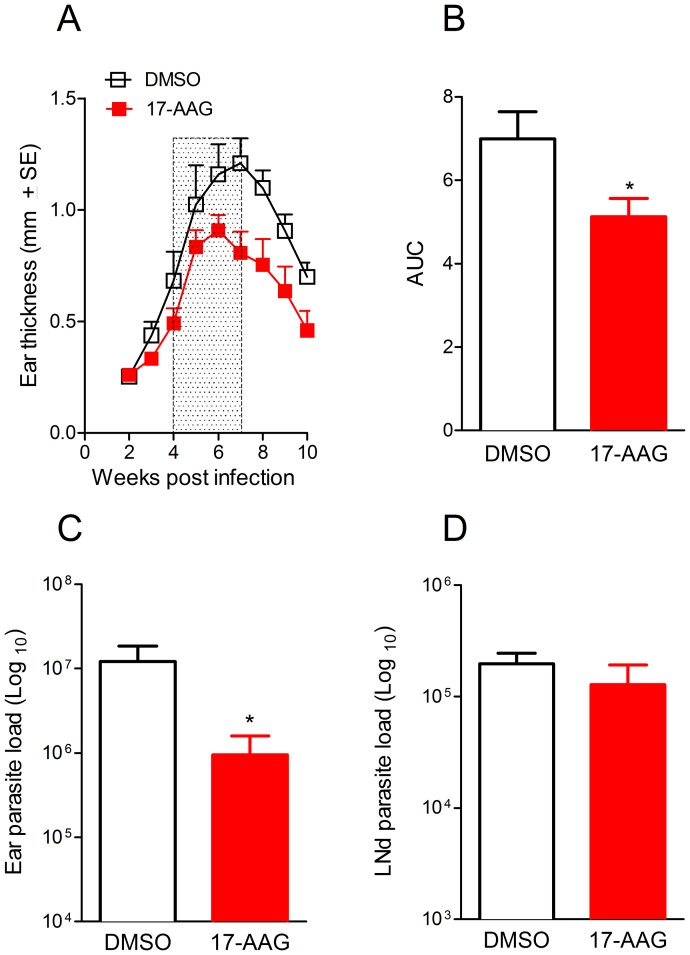
*In vivo* treatment with 17-AAG decreases *L. (V.) braziliensis* infection. Mice were infected with *L. (V.) braziliensis* and four weeks later, mice were treated with 17-AAG, 3x a week for 3 weeks (boxed area) or with vehicle (DMSO) alone. (A) The course of lesion development was monitored weekly. (B) Disease burden [shown as Area Under the Curves (AUC) depicted in (A)] in mice treated with 17-AAG or injected with DMSO. Parasite load was determined at the infection site (C) and at the dLN (D), 6 weeks later, by limiting dilution analysis. Data, shown as mean ±SEM, are from one of two independent repeats, each performed with 10 mice in each group (**p<0.01; *p<0.05).

Next, we tested the *in vivo* effect of 17-AAG against CL caused by *(V.) L. braziliensis*. These experiments were performed in an mouse model that reproduces aspects of the natural infection such as the presence of an ulcerated lesion, parasite dissemination to lymphoid areas and the development of a Th-1 type immune response [26]. BALB/c mice were inoculated with *L. (V.) braziliensis* in the ear dermis and lesion development was monitored weekly. Four weeks after infection, mice were treated with 17-AAG or with vehicle (DMSO) alone, three times a week, for three weeks. The ear thickness of mice treated with 17-AAG was significantly smaller compared to controls (Figure 5A). Disease burden, calculated by the area under the curve (AUC) obtained for the two experimental groups, was also significantly (*p*<0.05) different (Figure 5B), demonstrating the ability of 17-AAG to control lesion development, *in vivo*. 17-AAG treatment significantly (p<0.05) reduced the parasite load at the infection site, six weeks post inoculation (Figure 5C). However, treatment with 17-AAG was not able to reduce the parasite load within draining lymph nodes (dLN) (Figure 5D).

### Comparison of the *in vitro* killing effects of 17-AAG and amphotericin B

**Figure 6:**
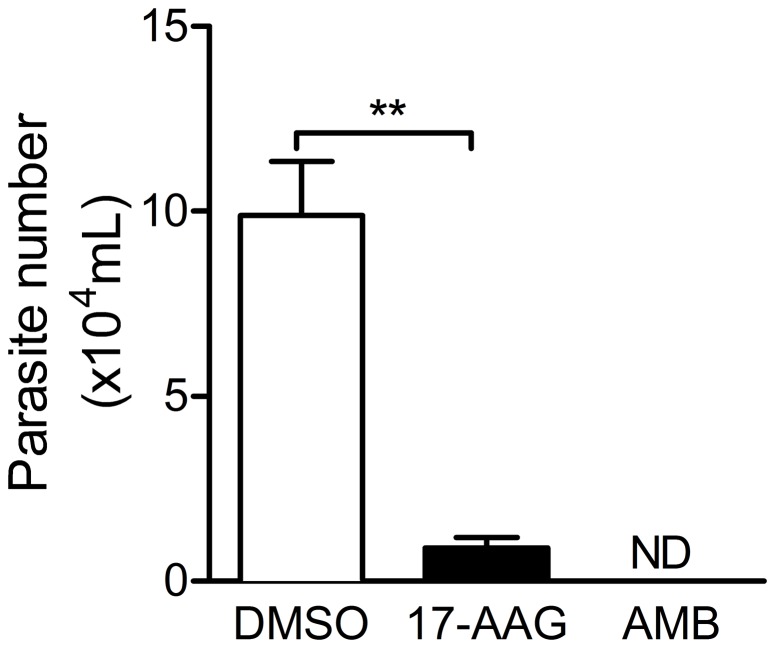
Comparison of the effects of 17-AAG and amphotericin B on the growth of intracellular *L. (V.) braziliensis*. *L. (V.) braziliensis-*infected macrophages were treated with 17-AAG or with amphotericin B (AMB) for 48 h. The number of viable parasites was evaluated by further culture in Schneider medium, free of 17-AAG. Data, shown as mean ±SEM, are from one of two independent repeats (***p<0.001). (ND, not detected).

In order to further characterize 17-AAG as an anti-leishmanial, its effect on infected macrophages was compared to that exerted by amphotericin B. Macrophages were infected with *L. (V.) braziliensis* and treated with 220 nM 17-AAG (IC_50_) or with amphotericin B (AMB) (0.27 µM). Forty-eight hours after medium replacement (Figure 6), cultures treated with either 17-AAG or amphotericin B displayed significantly lower parasite numbers (*p*<0.01) in comparison with controls, treated with vehicle (DMSO) alone.

## Discussion

HSP90 is a molecular chaperone fundamental for the life cycle of a variety or protozoa [31] and, as such, inhibitors of HSP90 have been suggested as novel chemotherapeutic agents against malaria [32], filariasis [33], [34] and schistosomiasis [34]. Recently, we showed that 17-AAG, a HSP90 inhibitor, reduced *L. (L.) amazonensis* infection *in vitro* [24]. Herein, we investigated the potential of 17-AAG as a chemotherapeutic agent against *L. (V.) braziliensis*, the main etiological agent of CL and MCL in Brazil [35]. We confirmed the effects of 17-AAG against this *L. (V.) braziliensis* promastigotes and we extended these findings to a pre-clinical model of CL.

Initially, we investigated the *in vitro* effects of 17-AAG, against both axenic promastigotes and intracellular amastigotes. Treatment of *L. braziliensis* promastigotes with the lower dose of 17-AAG (25 nM) already decreased promastigote viability. Herein, the IC_50_ determined for *L. (V). braziliensis* was comparable to that described for *L. (L). amazonensis* (65 nM) whereas in experiments performed with *L. (L). major,* the IC_50_ was established at 80 nM [24]. 17-AAG was equally effective at reducing intracellular amastigote numbers and the viability of surviving *L. (V). braziliensis* promastigotes. These effects were not associated with an increase in the microbicidal functions of macrophages as levels of NO, superoxide and TNF-α were diminished in the presence of 17-AAG. These results are in accordance with our previous report [24]. Additionally, the lack of amastigote replication in control macrophages could be attributed to innate microbocidal properties of macrophages that allow *L. (V). braziliensis killing*, as observed with *L. (V). guyanensis* and *L. (L.) major* [36]–[38]. In CL patients, an exacerbated inflammatory immune response is associated with the development of mucocutaneous leishmaniasis (rev. in [39]) whereas subclinical patients, who do not develop the disease, have a more controlled immune response [40]. Therefore, the possibility of selectively inducing parasite killing without contributing to overt inflammation is an important advantage for the treatment of CL using 17-AAG. Of note, macrophages treated with 17-DMAG alone displayed reduced production of IL-6, TNF-a and NO [41] whereas 17-AAG prevented iNOS expression upon stimulation with LPS or IFN-g [42].

Geldanamycin (GA), a HSP90 inhibitor analogous to 17-AAG, induces an anti-oxidative and attenuated inflammatory response in sepsis [43], autoimmune encephalitis [44], experimental atherosclerosis [45] and endotoxin-induced uveitis [46]. The proposed mechanism for these effects is the reduced nuclear translocation of NF-κB, reflecting in decreased production of IL-6, TNF-α and NO [41]. Although we cannot extrapolate the complexities of *in vivo* situations cited above, *L. (L.) amazonensis*-infected macrophages treated with 17-AAG displayed parasite killing, in spite of a diminished production of inflammatory mediators [24]. It has been shown that in BALB/c mice infected with *L. (V). braziliensis*, the density of INOS+ cells was higher when compared to *L. (L). amazonensis*-infected mice [47]. So, different responses to NO between *L. (V.) braziliensis* and *L. (L.) amazonensis* could also impact on the killing effect exerted by 17-AAG. In our previous work, [24], we showed that *L. (L) amazonensis* amastigotes displayed structural alterations following exposure of infected macrophages to 17-AAG. Visible alterations in the cytoplasm of parasites such as the presence of myelin figures, vesicles with double-layered membranes and mitochondrial segments inside membrane-bounded structures were the suggestive indications of autophagy, a process that naturally occurs in *Leishmania* and which plays an important role in the transition from promastigote to amastigote [48]. It is possible that inhibition of HSP90 activity interferes with cell cycle progression, blocking differentiation or expression of stage specific protein and, consequently, affecting survival in the intracellular environment.

17-AAG was also effective *in vivo*: mice infected with *L. (V.) braziliensis* and treated with 17-AAG showed a significantly smaller disease burden in parallel to a smaller parasite load at the infection site. However, 17-AAG was not able to alter parasite load at the draining lymph nodes (dLN), a site where *L. (V.) braziliensis* parasites persist following lesion healing [26]. In this experimental model, parasite persistence is associated with the presence of regulatory T cells (Tregs) that accumulate within dLNs of *L. (V.) braziliensis*-infected mice [49] and these Tregs control Th1 responses by IL-10-dependent mechanisms [50]. Although 17-AAG treatment controlled parasite replication at the infection site and promoted lesion healing, parasite persistence within distal sites such as the dLNs may have important effects with regards to maintenance of immunity to *Leishmania* [51] and/or development of mucotutaneous leishmaniasis, deserving further investigation.

Currently, the drugs available for the treatment of CL are limited and among them, pentavalent antimonials have been the choice for over 60 years. However, treatment is long (20-30 days), patients develop several side effects and, in the recent years, the number of cases refractory to treatment has increased [52], [53]. In the case of therapeutic failure, second-line drugs such as amphotericin B can be employed as well as combination of two available drugs [54]. Advantages of a combination treatment include increased efficacy, less drug resistance, lower drug dosage and a general decrease in side effects [55]. Herein, 17-AAG was as effective as amphotericin B at decreasing the parasite load within infected macrophages. Experimentally, the treatment of *L. infantum* and *L. panamensis* promastigotes with 17-AAG plus edelfosine improved the anti-leishmanicidal activity of the latter [56]. *In vitro* synergism was also observed for the combinations of paramomycin and amphotericin B against *L. (V.) braziliensis* [57]. *In vivo*, association of tamoxifen with amphotericin B yielded an additive effect in mice infected with *L. (L.) amazonensis* [58]. The combination of GA with fluconazole showed synergistic activity against *Candida albicans* isolates resistant to fluconazole alone [59]. Thus, we propose that combinations of 17-AAG and amphotericin B may be further investigated for the treatment of CL caused by *L. (V.) braziliensis*.

Herein, we reported that 17-AAG reduces *L. (V.) braziliensis* infection *in vitro* and *in vivo*. 17-AAG shows excellent bioavailability when given to mice by the i.p. route [60]. At 60 mg/kg, 17-AAG caused no changes in appearance, appetite, waste elimination, or survival of treated animals as compared to vehicle-treated controls. We employed with 20 mg/kg, a dose well below that reported as having any harmful effects, as those decribed by Solit et al. (equal or above 75 mg/kg) [61]. Given that HSP90 inhibitors, analogous to 17-AAG, have entered clinical trials with cancer patients [62], we propose that 17-AAG could be further investigated as a novel target for chemotherapy against cutaneous leishmaniasis.

## Supporting Information

Figure S1 **Determination of IC_50_ values for 17-AAG in** ***L. braziliensis*** **promastigotes.** Cells were treated in sextuplicate with 17-AAG for 48 hours with varying concentrations of 17-AAG. Following treatment, parasite viability was evaluated by direct counting. IC_50_ values (nM) were determined using GraphPad Prism.
(TIF)

Figure S2 **Treatment with 17-AAG controls** ***L. braziliensis*** **replication inside macrophages.** *L. braziliensis-* infected macrophages were treated with increasing concentrations of 17-AAG or with vehicle alone (DMSO). After 12–72 h, glass coverslips were stained with H&E and assessed for the percentage of infected macrophages (A) and the number of amastigotes per 100 macrophages (B) by light microscopy. Data, shown as mean ±SEM, are from one of three independent repeats (*p<0.05; **p<0.01 and ***p<0.001).
(TIF)

Figure S3 **Determination of IC_50_ values for 17-AAG in macrophages infected with** ***L. braziliensis*** **promastigotes.** Infected macrophages were treated in sextuplicate with 17-AAG for 72 hours with varying concentrations of 17-AAG. Following treatment, glass coverslips were stained with H&E and assessed for the presence of amastigotes by light microscopy. IC_50_ values (nM) were determined using GraphPad Prism.
(TIF)

Figure S4 **Cell viability following macrophage exposure to 17-AAG. Thioglycolate-elicited macrophages were exposed to with different concentrations of 17-AAG or to DMSO (vehicle) alone for 24 h.** Cell viability was evaluated by MTT assay.
(TIF)
