# Supplementary figures and images for: Chemotherapeutic Potential of 17-AAG against Cutaneous Leishmaniasis Caused by Leishmania (Viannia) braziliensis
Source: PLoS Negl Trop Dis. 2014 Oct 23;8(10):e3275. doi: 10.1371/journal.pntd.0003275 (PMC4207694; doi:10.1371/journal.pntd.0003275)

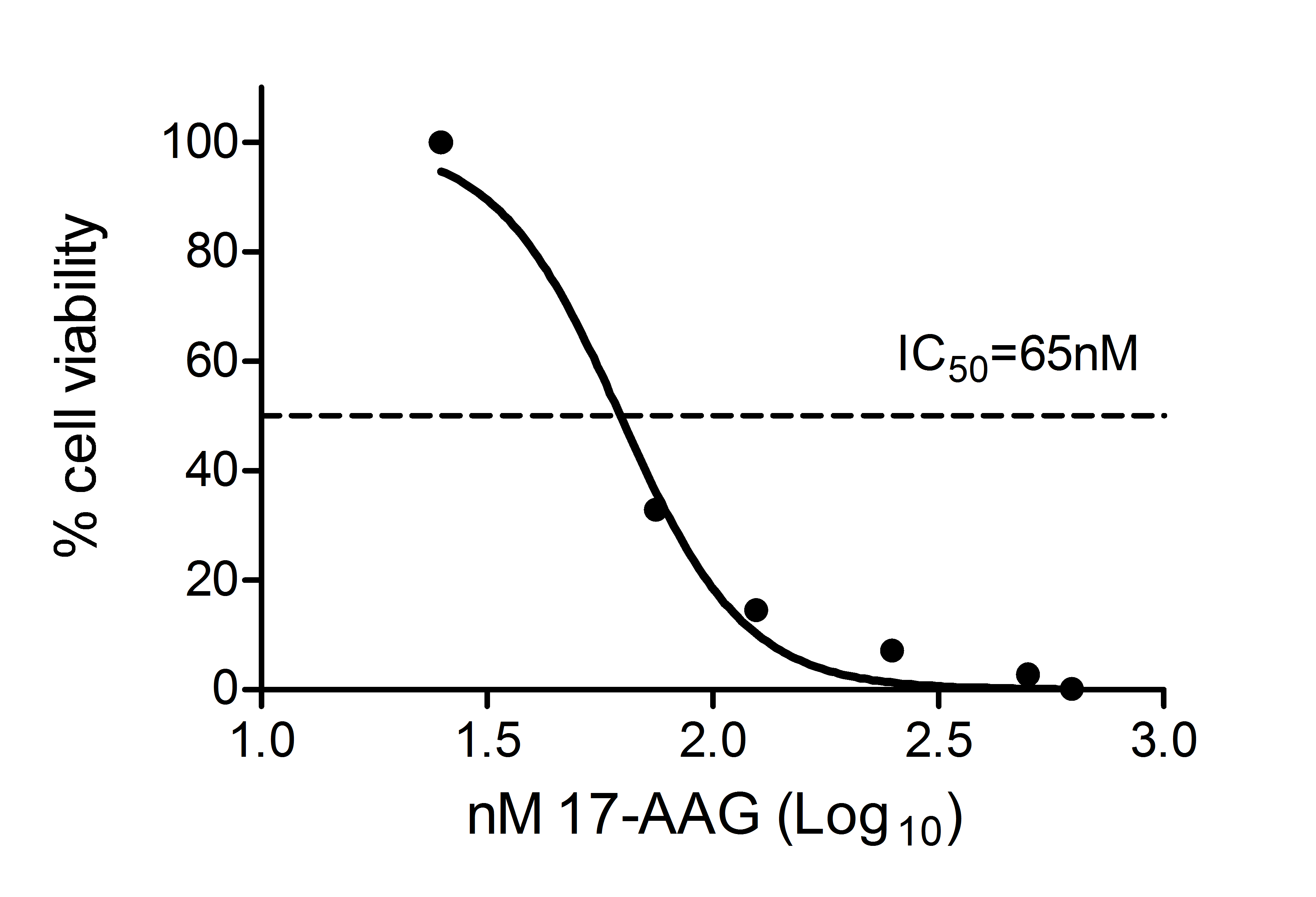

Supplement: Figure S1 — Determination of IC50 values for 17-AAG in L. braziliensis promastigotes. Cells were treated in sextuplicate with 17-AAG for 48 hours with varying concentrations of 17-AAG. Following treatment, parasite viability was evaluated by direct counting. IC50 values (nM) were determined using GraphPad Prism. (TIF) [file pntd.0003275.s001.tif]

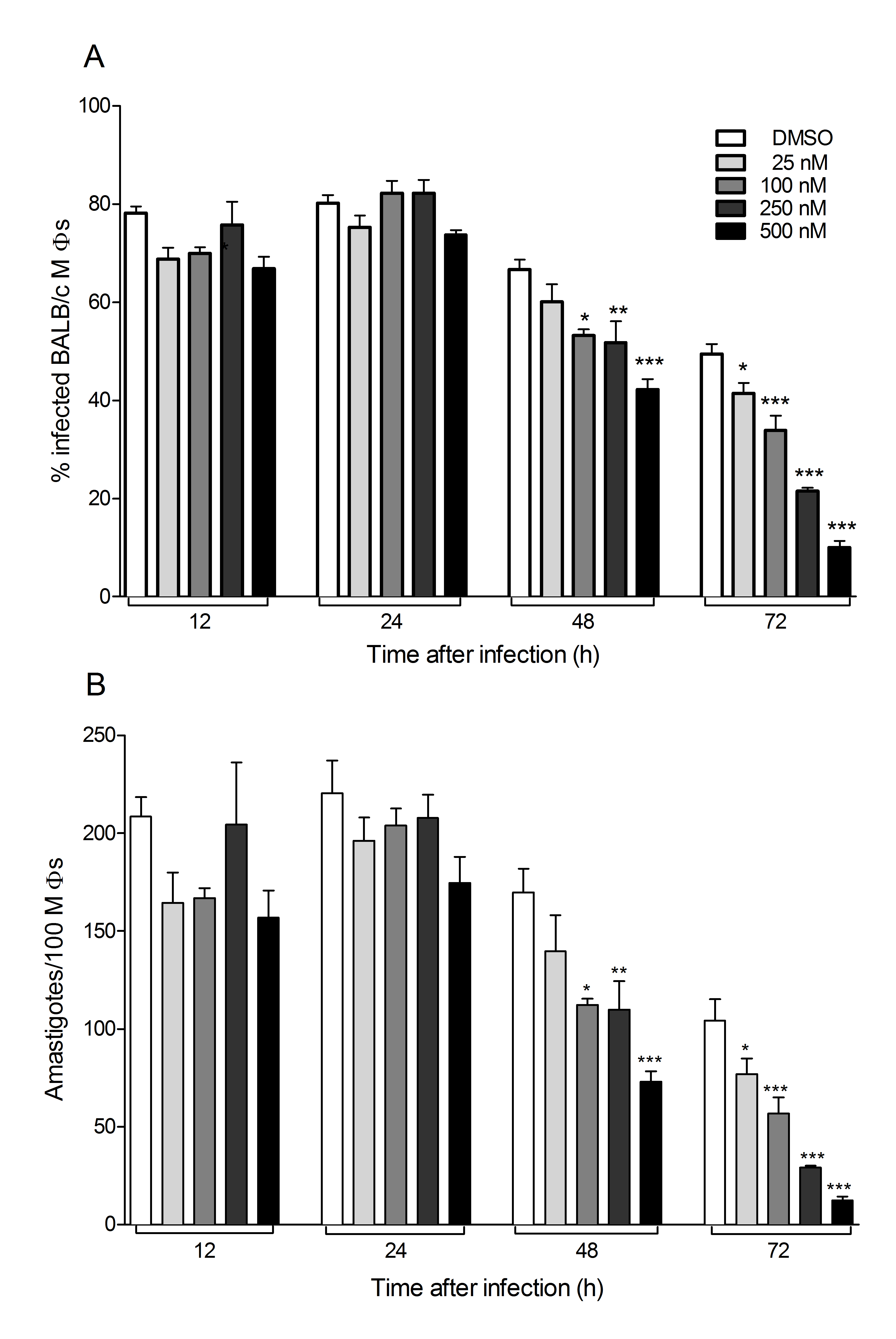

Supplement: Figure S2 — Treatment with 17-AAG controls L. braziliensis replication inside macrophages. L. braziliensis- infected macrophages were treated with increasing concentrations of 17-AAG or with vehicle alone (DMSO). After 12–72 h, glass coverslips were stained with H&E and assessed for the percentage of infected macrophages (A) and the number of amastigotes per 100 macrophages (B) by light microscopy. Data, shown as mean ±SEM, are from one of three independent repeats (*p<0.05; **p<0.01 and ***p<0.001). (TIF) [file pntd.0003275.s002.tif]

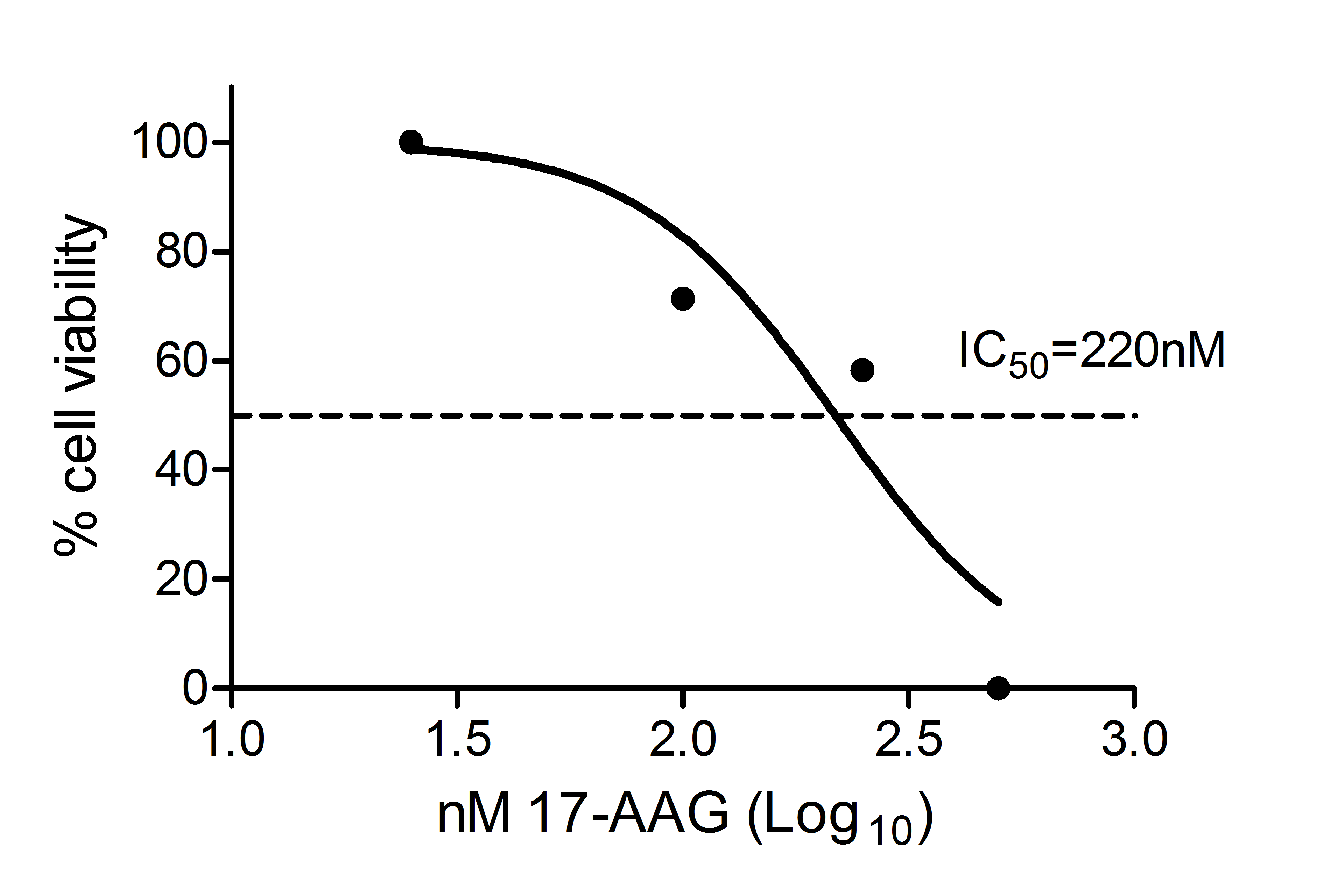

Supplement: Figure S3 — Determination of IC50 values for 17-AAG in macrophages infected with L. braziliensis promastigotes. Infected macrophages were treated in sextuplicate with 17-AAG for 72 hours with varying concentrations of 17-AAG. Following treatment, glass coverslips were stained with H&E and assessed for the presence of amastigotes by light microscopy. IC50 values (nM) were determined using GraphPad Prism. (TIF) [file pntd.0003275.s003.tif]

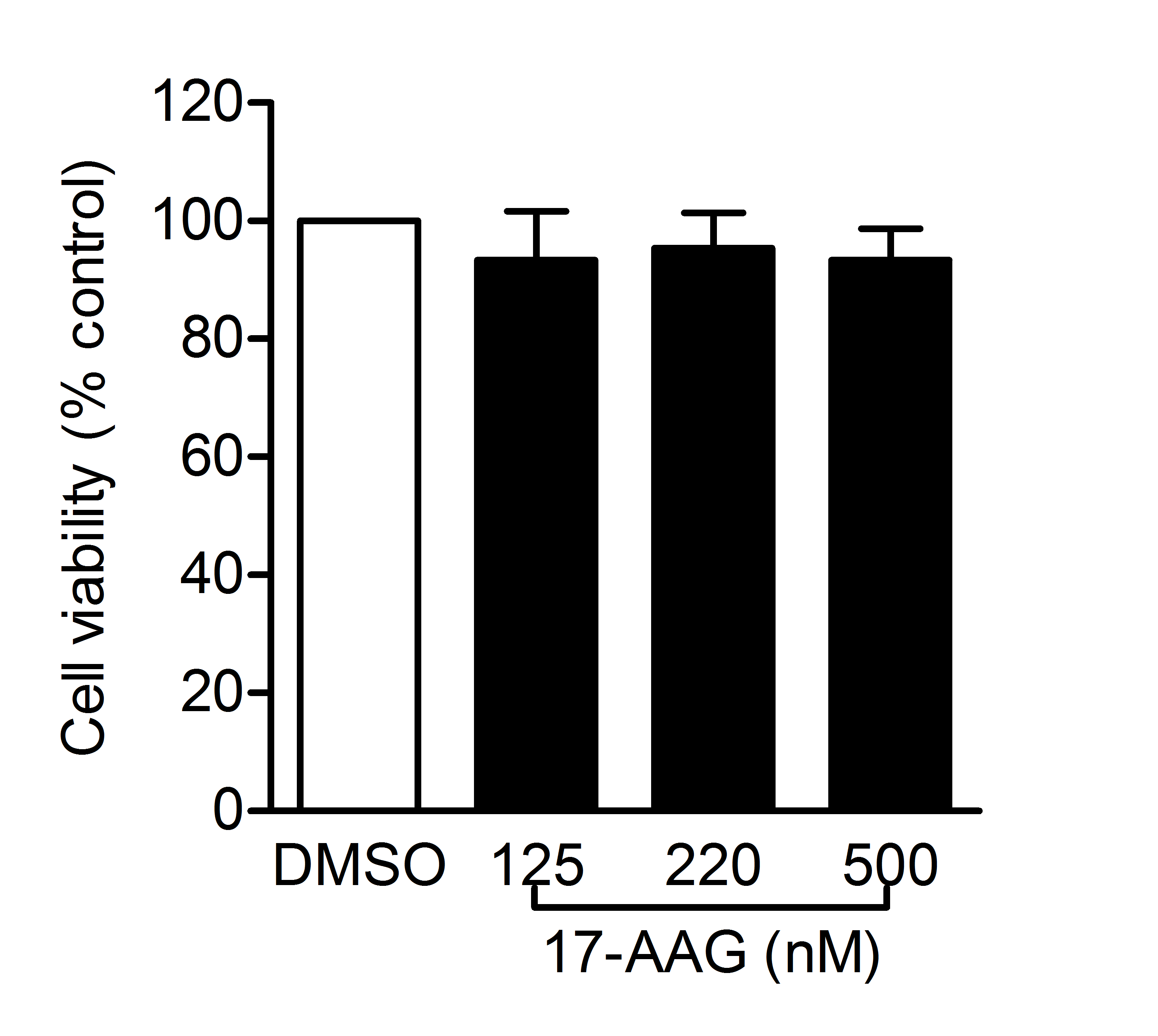

Supplement: Figure S4 — Cell viability following macrophage exposure to 17-AAG. Thioglycolate-elicited macrophages were exposed to with different concentrations of 17-AAG or to DMSO (vehicle) alone for 24 h. Cell viability was evaluated by MTT assay. (TIF) [file pntd.0003275.s004.tif]
